# Field testing primary stabbing headache criteria according to the 3rd beta edition of International Classification of Headache Disorders: a clinic-based study

**DOI:** 10.1186/s10194-016-0615-z

**Published:** 2016-03-11

**Authors:** Minwoo Lee, Min Kyung Chu, Juyoung Lee, Jinhyuk Yoo, Hong Ki Song

**Affiliations:** Department of Neurology, Kangdong Sacred Heart Hospital, Hallym University Medical Center, Seongan-ro 150, Gangdong-gu, Seoul 134-701 South Korea; Department of Neurology, Kangnam Sacred Heart Hospital, Hallym University, Seoul, South Korea

**Keywords:** Primary stabbing headache, Secondary headache, Headache, Criteria, ICHD-3 beta

## Abstract

**Background:**

The diagnostic criteria for primary stabbing headache (PSH) in the 3rd beta edition of International Classification of Headache Disorders (ICDH-3 beta) were recently revised. In the ICDH-3 beta, PSH is defined as short-lasting head pain spontaneous occurring as a single stab or series of stabs without autonomic symptoms and involving all head areas (i.e., not limited to the ophthalmic branch region of the trigeminal nerve). The aim of this study was to investigate the validity of the ICHD-3 beta criteria for PSH in a clinic-based setting.

**Methods:**

We prospectively collected data from patients with complaint of headache with stabbing pain without apparent cause at an initial visit to a secondary-care hospital from March 2009 to March 2014. Patients were followed up for 2 weeks to assess changes in clinical characteristics and secondary causes of pain.

**Results:**

Data from 280 patients with headache with stabbing pain without apparent cause were collected, and 245 patients were followed up for 2 weeks. Secondary causes for stabbing headache were observed in 9 patients (herpes zoster in 7 patients and Bell’s palsy in 2 patients) after 2 weeks. The remaining 236 patients fulfilled the diagnostic criteria for PSH according to ICHD-3 beta. Only 22 patients met the diagnostic criteria for PSH according to ICHD-2.

**Conclusions:**

All patients with headache with stabbing pain without cranial autonomic symptoms fulfilled the diagnostic criteria for PSH according to ICHD-3 beta at the initial visit. Secondary causes for headache with stabbing pain were revealed in a small proportion (3.7 %) of patients after 2 weeks of follow-up.

## Background

Stabbing pain in the head that lasts a few seconds is a common type of headache [1–4]. The second edition of the International Classification of Headache Disorders (ICHD-2) proposed diagnostic criteria for this type of headache, which is known as primary stabbing headache (PSH) (code 4.1). These criteria require that head pain occurs exclusively or predominantly in the first division of the trigeminal nerve without apparent cause [5]. Previous studies have shown, however, that PSH can occur outside the trigeminal region, including in the extracephalic regions. The third beta edition of the International Classification of Headache Disorders (ICHD-3 beta), therefore, has revised the diagnostic criteria for PSH (code 4.1) as head pain that occurs spontaneously as a single stab or series of stabs without cranial autonomic symptoms in all head regions [1–4, 6–12].

The diagnostic criteria for PSH according to ICHD-3 beta have not been validated yet. The purpose of this study was 1) to test the validity of the diagnostic criteria for PSH according to ICHD-3 beta; 2) to investigate the clinical characteristics of PSH according to pain location, which represents a criterion that changed from ICHD-2 to ICHD-3 beta; and 3) to investigate the clinical characteristics of secondary stabbing headache (SSH) at a follow-up visit among patients diagnosed with PSH at an initial visit.

## Methods

### Participants

We consecutively recruited patients with short-duration headache without apparent cause who visited the Department of Neurology at Kangdong Sacred Heart Hospital, a secondary-care hospital in Korea, from March 2009 to March 2014. Exclusion criteria included 1) 19 years old or younger; 2) patients with possible causes for stabbing headache; and 3) patients who refused to participate the study. After 5 years of consecutive recruitment, we analyzed data of structured interview and medical records of all the subjects in accordance with the ICHD-3 beta diagnostic criteria of PSH in the present study. This study was approved by the Institutional Review Board of Kangdong Sacred Heart Hospital. Written informed consent was obtained from all participants.

### Assessment

A physician (HKS) interviewed all patients using a structured questionnaire and performed the physical examination. The information collected included age; gender; headache duration; location, intensity, frequency, and quality of pain; preceding infection; presence of allodynia; associated symptoms; and recurrence of stabbing headache. Patients were followed up for 2 weeks to investigate response to treatment, change in clinical characteristics, and secondary causes for stabbing headache.

The location of pain was recorded relative to the trigeminal and upper cervical regions. The trigeminal region was subdivided into ophthalmic (V1), maxillary (V2), and mandibular (V3) branches. The upper cervical region was subdivided into the lesser occipital nerve (LON), greater occipital nerve (GON), and greater auricular nerve.

A headache specialist (HKS) treated all patients with PSH. Patients with PSH were treated with gabapentin (300 mg bid), naproxen (275 mg bid), or amitriptyline (5 mg hs) based on the judgement of the specialist. Each patient was categorized as either a responder (cessation of stabbing pain) or non-responder (continuation of stabbing pain) in response to treatment after 2 weeks. After 2 weeks of follow up, we asked our participants to inform us if stabbing headache persisted more than 1 week or occurring other secondary causes of stabbing headache to detect persisting symptoms or late-onset SSH.

### Statistics

Data were analyzed using SPSS Statistics for Windows, Version 21 (Armonk, New York, USA). Student’s t-tests, chi-square tests, and Mann–Whitney U tests were used for comparison as appropriate. A p-value < 0.05 was considered statistically significant

## Results

### Subjects

**Fig. 1:**
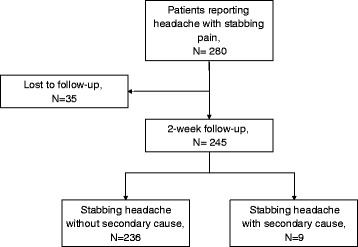
Flow chart of the number of participants at each stage of the study

During the study period, a total of 280 patients with stabbing headache were recruited during the initial visit. Of these patients, 243 were prescribed medication for their stabbing headache at the initial visit and 245 were followed for 2 weeks (Fig. 1) *Clinical characteristics at initial visit*.

**Fig. 2:**
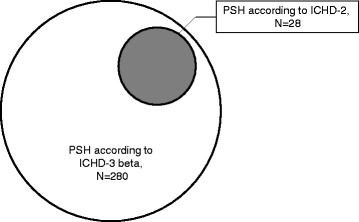
Distribution of participants diagnosed with primary stabbing headache at the initial visit according to ICHD-3 beta and ICHD-2 criteria. PSH: primary stabbing headache; ICHD-2: the second edition of the international classification of headache disorders; ICHD-3 beta: the third beta edition of the international classification of the headache disorders

Of the 280 patients with stabbing headache without apparent cause at the initial visit, 103 were men and 177 were women. The mean age of patients was 51.4 ± 13.3 years. The mean duration from the onset of symptoms to the initial visit was 4.0 ± 3.7 days. The mean visual analog scale (VAS) score for headache intensity was 4.6 ± 1.6. Seventy-four (26.4 %) patients reported mild pain (VAS score 1–3), 165 (58.9 %) reported moderate pain (VAS score 4–6), and 41 (14.6 %) reported severe pain (VAS score 7–10). The duration of a single stab was within 1–2 s in 262 patients (93.6 %) and 3–5 s in 18 patients (6.4 %). No patient reported autonomic symptoms during their headache attacks. There was no predominant pain lateralization (145 on the left, 133 on the right, and 2 on both the left and right). Pain was reported in the LON region in 129 patients, GON region in 80 patients, V1 region of the trigeminal nerve in 27 patients, V2 region in 28 patients, and V3 region in 8 patients. One patient had pain in both the LON and GON, and another patient had pain in both V1 and V2. No patients reported stabbing headache in both the trigeminal and upper cervical regions. A total of 243 patients (86.8 %) were treated with medication. Fifty-three patients (18.9 %) reported allodynia on the same side of the stabbing headache. Allodynia was reported in 8 patients who reported stabbing headache in the V1 region (Table 1).

**Table 1:** Clinical characteristics of patients with stabbing headache according to ICHD-3 beta criteria at the initial and follow-up visit

		All patients^a^ (N = 280)	Primary stabbing headache^b^ (N = 236)	Secondary stabbing headache^b^ (N = 9)	P-value
Gender					
	Male	103 (36.8 %)	89 (37.7 %)	5 (55.5 %)	0.280
	Female	177 (63.2 %)	147 (62.3 %)	4 (44.4 %)	
Mean age		51.4 ± 13.3	50.6 ± 13.4	54.6 ± 14.1	0.385
Fixed location			230 (97.5 %)	9 (100 %)	0.628
Pain lateralization					
	Unilateral	278 (99.3 %)	234 (99.2 %)	9 (100.0 %)	0.781
	Bilateral	2 (0.7 %)	2 (0.8 %)	0 (0.0 %)	
Location of pain					
	V1 region	27 (9.6 %)	22 (9.3 %)	1 (11.1 %)	0.857
	V2 region	28 (10.0 %)	27 (11.4 %)	1 (11.1 %)	
	V3 region	8 (2.9 %)	7 (3.0 %)	0 (0.0 %)	
	All trigeminal regions	63 (22.5 %)	55 (23.3 %)	2 (22.2 %)	0.940
	Lesser occipital nerve region	129 (46.1 %)	109 (46.2 %)	4 (44.4 %)	
	Greater occipital nerve region	80 (28.6 %)	64 (27.1 %)	3 (33.3 %)	
	Great auricular nerve	6 (2.1 %)	6 (2.5 %)	0 (0.0 %)	
	All upper cervical regions	217 (77.5 %)	181 (76.7 %)	7 (77.8 %)	0.891
	Multiple locations	2 (0.7 %)	2 (0.8 %)	0 (0.0 %)	0.782
Visual Analog Scale for pain intensity		4.6 ± 1.6	4.5 ± 1.6	6.8 ± 1.6	<0.001
Onset to hospital visit interval (days)		4.0 ± 3.7	4.0 ± 3.9	5.1 ± 2.4	0.341
Improvement at follow-up visit			226 (95.8 %)	5 (55.5 %)	<0.001
Duration of improvement (days) among responders			3.2 ± 1.9	4.8 ± 1.5	0.060
Preceding infection		21 (7.5 %)	21 (8.9 %)	0 (0.0 %)	0.083
Stressful event		61 (21.8 %)	48 (20.3 %)	4 (7.7 %)	0.083
Allodynia		53 (18.9 %)	45 (19.1 %)	2 (22.1 %)	0.814

^a^Assessed at initial visit

^b^Assessed at follow-up visit

ICHD-3 beta: the third beta edition of the International Classification of Headache Disorders. V1: ophthalmic branch of trigeminal nerve; V2: maxillary branch of trigeminal nerve; V3 mandibular branch of trigeminal nerve

### Clinical characteristics at follow-up

Of the 245 patients who were followed for 2 weeks after the initial visit, 231 (94.3 %) reported improvements. Mean duration for improvement among the responder group was 3.2 ± 1.9 days. Secondary causes for stabbing headache were observed in 9 patients (3.8 %) (herpes zoster in 7 patients and Bell’s palsy in 2 patients); these patients were initially diagnosed with PSH (Table 1). Improvement at follow-up was not significantly different between those who did and did not take medication (189/199 vs. 37/37, respectively; *p* = 0.164). Among 10 PSH patients with persisting stabbing headache at follow-up, no patient additionally visited for their headaches. All 4 SSH patients with persisting stabbing headache at follow-up were diagnosed as having herpes zoster. Three of them improved their stabbing headache after 1 week and the remaining 1 patient improved after 2 weeks.

### Validating the ICHD-3 beta criteria for PSH

All 280 patients who participated in the present study fulfilled the diagnostic criteria for PSH at the initial visit. Of the 245 patients who were followed for 2 weeks, 9 (3.2 %) did not meet the criteria for PSH at the second visit due to secondary causes for their stabbing headaches. These patients were diagnosed with SSH. Thus, at follow-up, 236 patients were diagnosed with PSH according to ICHD-3 beta. In contrast, only 28 patients (10.0 %) fulfilled the ICHD-2 criteria for PSH (Fig. 2). The remaining 252 patients were diagnosed with trigeminal neuralgia (N = 7, 2.5 %) or unclassified headache (N = 245, 87.5 %) based on ICHD-2 criteria. One patient (4.5 %) who was initially diagnosed with PSH according to ICHD-2 criteria was diagnosed with SSH due to Bell’s palsy at follow-up. The proportion of patients with PSH re-diagnosed with SSH at follow-up was not significantly different between ICHD-3 beta and ICHD-2 criteria (9/271 vs. 1/27, *p* = 0.919). No patient was classified as having probable stabbing headache (code 4.7.1) at initial and follow-up visit. The number of patients with PSH re-diagnosed with SSH at follow-up was not significantly different between ICHD-3 and ICHD-2 criteria.

### Clinical characteristics of PSH and SSH

We compared demographic and headache characteristics between PSH and SSH. There was no significant difference in gender, age, location of pain, preceding infection, or stressful events between patients with PSH and patients with SSH. VAS for pain was significantly higher in patients with SSH than patients with PSH. Improvement in pain at follow-up was more common in patients with PSH than patients with SSH. The duration of improvement among responders was longer in SSH than PSH (Table 1). However, further follow up of patients with SSH revealed favorable outcome within one month of onset.

### Clinical characteristics of PSH involving V1 compared with other regions

Demographic and headache characteristics such as age, gender, VAS for pain, improvement after 2 weeks, duration of improvement among responders, preceding infection, stressful events, and allodynia were not significantly different between patients with PSH who reported V1 region involvement and those who reported involvement of regions other than V1 except stressful event history. Stressful event history was more common among subjects with PSH involving other than V1 region (Table 2).

**Table 2:** Clinical characteristics of primary stabbing headache by ICHD-3 beta criteria at initial visit according to the pain location between V1 and other than V1

	V1 region^a^ N = 28	Other than V1 region^b^ N = 252	P-value
Gender			
Male	13 (46.4 %)	90 (35.7 %)	0.265
Female	15 (53.6 %)	162 (64.3 %)	
Age	50.5 ± 13.4	51.5 ± 13.3	0.912
Visual analog scale for pain intensity	4.4 ± 1.3	4.7 ± 1.6	0.160
Improvement at follow-up^c^	22 (100.0 %)	204 (95.3 %)	0.301
Mean duration of improvement (days) among responders	4.1 ± 3.1	3.5 ± 2.7	0.135
Preceding infection	4 (14.2 %)	17 (6.7 %)	0.151
Stressful event history	2 (7.1 %)	59 (23.4 %)	0.048
Allodynia	8 (28.5 %)	45 (17.8 %)	0.170

^a^One patient had stabbing headache in both the V1 and V2 regions

^b^V2, V3, lesser occipital nerve, greater occipital nerve, great auricular nerve, and multiple dermatomes. One patient with multiple dermatomes, including V1, were included

_c_22 out of 28 patients with pain at V1 region and 216 out of 252 patients with pain at other than V1 regions were followed at 2 weeks after initial visit, respectively

ICHD-3 beta: the third beta edition of the International Classification of Headache Disorders. V1: ophthalmic branch of trigeminal nerve; V2: maxillary branch of trigeminal nerve; V3 mandibular branch of trigeminal nerve

### Clinical characteristics of PSH involving trigeminal area and cervical area

We also compared clinical characteristics at initial visit between PSH involving trigeminal area and cervical area. Stressful event history was significantly higher among PSH subjects involving cervical area than those involving trigeminal area (Table 3).

**Table 3:** Clinical characteristics of primary stabbing headache by ICHD-3 beta criteria at initial visit according to the pain location between trigeminal and cervical areas

	Trigeminal area^a^ N = 55	Cervical area^b^ N = 225	P-value
Gender			
Male	23 (41.8 %)	80 (35.5 %)	0.388
Female	32 (58.9 %)	145 (64.5 %)	
Age	49.5 ± 13.4	51.8 ± 13.2	0.968
Visual analog scale for pain intensity	4.4 ± 1.6	4.7 ± 1.6	0.787
Improvement at follow-up	43 (97.8 %)	188 (97.9 %)	0.937
Mean duration of improvement (days) among responders^c^	3.8 ± 3.2	3.4 ± 2.7	0.145
Preceding infection	5 (10.9 %)	16 (7.1 %)	0.617
Stressful event history	6 (6.8 %)	55 (24.4 %)	0.029
Allodynia	14 (25.4 %)	39 (17.3 %)	0.168

^a^V1, V2 and V3 dermatomes

^b^Lesser occipital nerve, greater occipital nerve, great auricular nerve, and multiple dermatomes. One patient with multiple dermatomes, including V1 region, were included

^c^44 out of 55 patients with pain at trigeminal area and 192 out of 225 patients with pain at cervical area were followed at 2 weeks after initial visit, respectively

ICHD-3 beta: the third beta edition of the International Classification of Headache Disorders

## Discussion

The key findings in the present study are as follows: 1) the diagnostic criteria for PSH in ICHD-3 beta enabled diagnosis of primary headaches with stabbing pain as PSH, most of which were not classified according to ICDH-2 criteria; 2) a small proportion (2.8 %) of new-onset stabbing headaches, which were initially diagnosed as PSH, had secondary causes that were revealed at 2-week follow-up; 3) pain intensity was more severe and pain improvement was slower among patients with SSH than that in patients with PSH.

This is the first study to field test the ICHD-3 beta diagnostic criteria for PSH. In the present study, all 280 subjects with short-lasting stabbing headache without apparent cause and without autonomic symptoms at the initial visit fulfilled the diagnostic criteria for PSH according to ICHD-3 beta. In contrast, only 9.6 % of patients met the criteria for PSH according to ICHD-2. This finding suggests that most primary stabbing headaches without cranial autonomic symptoms fulfill the diagnostic criteria for PHS.

One important change in the diagnostic criteria for PSH in ICHD-3 beta from ICHD-2 was the definition of pain location. The diagnostic criteria for PSH in ICHD-3 beta included all head regions, whereas criteria in ICHD-2 included the V1 region only [12]. To investigate the clinical characteristics of PSH according to pain location, we compared patients with PSH involving the V1 region to patients with PSH involving non-V1 regions. Age, gender, pain intensity, improvement of pain, duration of improvement among responders, preceding infection, stressful events, and allodynia were not significantly different according to pain location. These findings suggest that all clinical characteristics of PSH except pain location are similar between ICHD-3 beta and ICHD-2.

Another change in ICHD-3 beta criteria from ICHD-2 criteria was the definition of accompanying symptoms. Diagnostic criteria for PHS in ICHD-2 included as a criterion that no accompanying symptoms were present [5]. According to ICHD-2, PSH was excluded as a diagnosis if stabbing headache without apparent cause was accompanied by symptoms such as nausea, vomiting, photophobia, or phonophobia [1, 5]. In ICHD-3 beta, this criterion was revised to a lack of “cranial autonomic symptoms” [12]. However, we did not investigate accompanying symptoms except allodynia, and no patient reported cranial autonomic symptoms in the present study. This change in the definition of accompanying symptoms in ICHD-3 beta may have excluded fewer primary headaches with stabbing pain as PSH than ICHD-2.

PSH has been considered responsive to indomethacin [13–16]. Responsiveness to indomethacin was described in the “*Comments*” of ICHD-1 and ICHD-2 [5, 17]. However, responsiveness to indomethacin was removed from ICHD-3 beta [12]. In the present study, no patient was treated with indomethacin, and most patients with PSH improved at follow-up. Mean duration for improvement was 3.2 days among responders. In addition, improvement at follow-up was not significantly different between patients who did and did not take medication. These findings suggest that most patients with PSH have a good prognosis; specific responsiveness to indomethacin was difficult to conclude.

Our study, which followed patients who were initially diagnosed with PSH, revealed that a small but significant proportion of patients were found to have SSH at follow-up. Previous studies have reported secondary causes for stabbing headache, including Bell’s palsy, herpes zoster, cerebral infarction, transient ischemic attack, post-infection, thalamic hemorrhage, autoimmune diseases, pituitary tumor, Chiari Malformation Type 1, C1-C2 subluxation, meningioma, and multiple sclerosis [2, 6, 15, 18–24]. We compared clinical characteristics of PSH and SSH and found no significant differences except in pain intensity. This finding suggests that the risk for SSH is higher for a PSH-diagnosed stabbing headache with severe pain intensity. The risk for SSH was not significantly different between ICHD-3 beta and ICHD-2 criteria in the present study.

Bell’s palsy and herpes zoster have been reported as common causes of secondary stabbing headache [6, 24]. Retroauricular stabbing pain in Bell’s palsy often presents 2–3 days before the onset of facial weakness [25, 26]. Herpes zoster is usually accompanied by short-lasting stabbing pain in the involved sites. Pain in herpes zoster often presents 4–5 days before the presentation of skin lesions [26]. Most stabbing headaches are reported to be improved within 2 weeks [1, 2]. Given these findings, we followed patients with PSH for 2 weeks. However, stabbing headache persisted in some patients, and some SSH may have been classified as PSH in the present study.

Our study has several limitations. First, we did not perform nerve block or examine tenderness in the occipital area to diagnosis occipital neuralgia (ON). According to ICHD-3 beta, ON pain is associated with tenderness over the affected nerve branch or trigger points at the emergence of the greater occipital nerve or C2 distribution [12]. Temporary relief of the pain by local anesthetic block is also required. ON in the GON and LON areas may have been diagnosed as PSH in the present study. Second, not all patients were treated, and treatment medication was not the same across patients. In the present study, a headache specialist decided which treatment to administer to each patient based on his judgement. The rate and duration of improvement was similar between patients who did and did not receive treatment. This finding is consistent with previous studies in which PSH is reported to be an easily treatable headache disorder [1, 2]. Third, we did not investigate prior history of migraine or other primary headache disorders. Though rather speculative, previous studies reported that 21 - 38 % of patients with primary stabbing headache had history of migraine [2, 16].

The strengths of this study are its large sample size, 2-week follow-up to investigate changes in clinical characteristics and secondary causes, comparisons of diagnostic criteria between ICHD-3 beta and ICHD-2, and headache diagnosis by a headache specialist, which may avoid inter-examiner bias. Future work will include a longer duration of follow-up and exclusion of ON via an examination of tenderness and performance of a nerve block.

## Conclusion

In summary, the diagnostic criteria for PSH according to ICHD-3 beta are valid and enable the diagnosis of most primary headaches with stabbing pain, which were not classified according to ICDH-2. A small proportion of participants with PSH according to ICHD-3 beta criteria at the initial visit were re-diagnosed with SSH at 2-week follow-up owing to secondary causes. This information may provide a better understanding of PSH and it diagnostic criteria according to ICHD-3 beta.

### Ethical standards

The study was approved by the Institutional Review Board/ethics committee of Hallym University Kangdong Sacred Heart Hospital and was performed in accordance with the ethical standards laid down in the 1964 Declaration of Helsinki and its later amendments. Written informed consent was obtained from all participants.

## Abbreviations

GON: greater occipital nerve
ICHD-2: International Classification of Headache Disorders, 2nd edition
ICHD-3 beta: International Classification of Headache Disorders, 3rd edition (beta version)
LON: lesser occipital nerve
ON: occipital neuralgia
PSH: primary stabbing headache
SSH: secondary stabbing headache
